# Non-destructive monitoring of forming quality of self-piercing riveting via a lightweight deep learning

**DOI:** 10.1038/s41598-023-32827-7

**Published:** 2023-04-13

**Authors:** Sen Lin, Lun Zhao, Sen Wang, Md Shafiqul Islam, Wu Wei, Xiaole Huo, Zixin Guo

**Affiliations:** 1 Institute of Intelligent Manufacturing Technology, Shenzhen Polytechnic, Shenzhen, 518055 China; 2 Faculty of Mechanical and Electrical Engineering, Kunming University of Science and Technology, Kunming, 650500 China; 3 Department of Mechanical Engineering, Blekinge Institute of Technology, 37179 Karlskrona, Sweden; 4 School of Mechanical Engineering, Guizhou University, Guiyang, 550025 China

**Keywords:** Mechanical engineering, Structural materials

## Abstract

Self-piercing riveting (SPR) has been widely used in automobile body jointing. However, the riveting process is prone to various forming quality failures, such as empty riveting, repeated riveting, substrate cracking, and other riveting defects. This paper combines deep learning algorithms to achieve non-contact monitoring of SPR forming quality. And a lightweight convolutional neural network with higher accuracy and less computational effort is designed. The ablation and comparative experiments results show that the lightweight convolutional neural network proposed in this paper achieves improved accuracy and reduced computational complexity. Compared with the original algorithm, the algorithm’s accuracy in this paper is increased by 4.5$\%$, and the recall is increased by 1.4$\%$. In addition, the amount of redundant parameters is reduced by 86.5$\%$, and the amount of computation is reduced by 47.33$\%$. This method can effectively overcome the limitations of low efficiency, high work intensity, and easy leakage of manual visual inspection methods and provide a more efficient solution for monitoring the quality of SPR forming quality.

## Introduction

**Figure 1:**
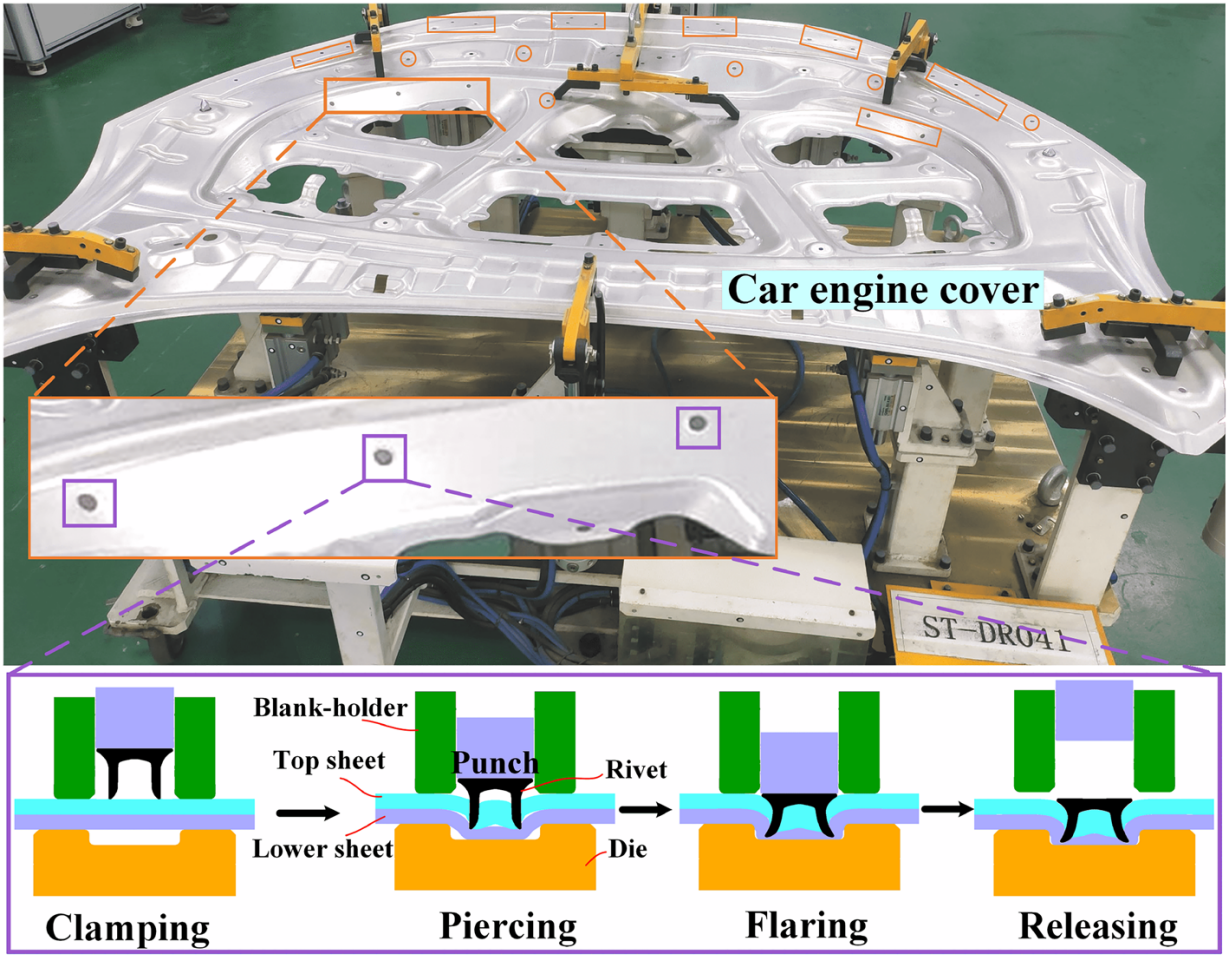
Body SPR forming and SPR technology.

**Figure 2:**
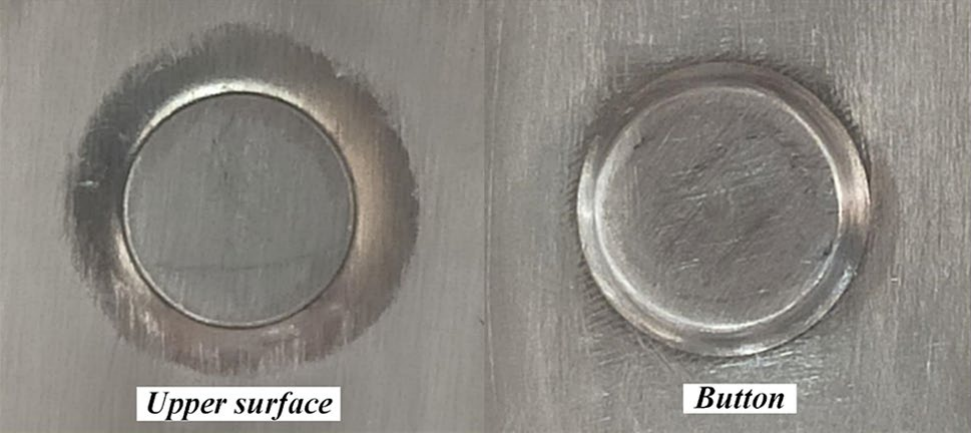
Front and back side of the formed qualified joint.

Self-piercing riveting, as a new, green and efficient jointing process, can achieve the similar, dissimilar, double and multi-layer thin sheet material jointing^1–3^. Compared with the traditional thin sheet material connection method, the SPR process has many advantages, such as: the fast connection process, no pre-forming holes and pre-treatment, the green and efficient forming process, no production of waste, sparks and other harmful substances. In addition, it is easy to integrate into the robot arm, which can realize automated mass production. SPR joints have excellent mechanical properties and sealing, low equipment maintenance costs, and long service life of riveting tools, which is up to more than 200,000 times^4–6^. Based on the above advantages, SPR has become one of the most important processes to achieve lightweight automobile body. According to statistics, the number of body rivets on Audi Q7 is 2855. The riveting point on Jaguar XFL body is 2754^7^, about 1300 on Lamborghini Gallardo body^8^, 1235 on Mercedes-Benz SL (R231) body is, and 357 on Cadillac CT6 body^9,10^. The body SPR molding effect and the SPR molding process flow are shown in Fig. 1. The molding process of SPR is divided into four stages. The first stage is clamping, which aligns the sheets to be riveted, and the SPR system controls the crimping ring to press the sheets, that is, the preload force is applied to the sheets to fix the sheets to be riveted. The second stage is piercing, in which the rivet is quickly pushed through the upper sheet and into the lower sheet by the action of the punch. The third stage is flaring, that is, the rivet continues to pierce the lower sheet by the action of the punch, and the rivet leg gradually extends outward by the action of the bottom mold and the sheet, thus forming a strong and reliable mechanical interlock. The fourth stage is releasing, after the punch reaches the set stroke or reaches the mechanical limit, it stops working and releases after holding the pressure. The entire forming process of the SPR joint is monitored by the curve tolerance band method^11^ and manual visual inspection of the forming quality of the joint. The appearance of the formed joint is shown in Fig. 2. The appearance of the qualified joint requires an accurate riveting position, no puncture, full button, and no cracks. In addition, the force-displacement curve is within the tolerance zone during the forming process.

At present, the research progress in the field of SPR forming quality monitoring is slow, and the existence of a large number of riveting points also puts forward new requirements for SPR appearance quality monitoring. Currently, the conventional destructive section method, curve tolerance zone method^11^, and human visual appearance quality inspection are mainly used to check the quality of SPR. Except for the tolerance zone method , which can detect the molding process of all joints, the other methods described above are not suitable for large-scale rapid inspection, often for sampling inspection. At present, it is widely acknowledged that the more dependable quality monitoring approach is still the tolerance zone method, that is, the tolerance band of the qualified joint is generated by the force-displacement curve and offset of the formed qualified joint. The forming joint beyond the tolerance band is considered unqualified, and the tolerance band method is not only insensitive to lateral displacement but also unable to monitor defects such as rivet offset, sheet cracking, button cracking, button drop, etc., and there is a large detection blind spot. These defects are then often a key factor affecting the reliability of the joint. Therefore, the monitoring of the forming quality of SPR molding is a difficult problem, and how to build a comprehensive and efficient SPR quality inspection system to improve the reliability and practicability of the SPR process needs further research.

In recent years, with the rapid development of artificial intelligence algorithms, and the accumulation of raw data in various disciplines is increasing. Deep learning algorithms are now widely employed in mechanical^12^, biomedical^13,14^, civil engineering^15,16^, materials^17^, and other sectors^18^, allowing for more efficient problem-solving in a variety of fields. In the field of mechanical smart manufacturing, digital twins are combined with reinforcement learning to extend to more complex manufacturing systems, using deep neural networks to solve the challenges posed by large state and action spaces^19^. In the field of biomedicine, the pathological diagnosis and analysis of medical images can be effectively realized through deep learning algorithms^20^; In the monitoring of structure health, combined with the full convolutional network algorithm to build a crack detection system for steel structures, long-term and effective monitoring of cracks in steel structures can be effectively realized^21^. The effective prediction of material structure design and performance can be realized by deep learning^22,23^. And the SPR process combined with artificial neural network (ANN) can quickly achieve parameter optimization. Through SPR technology combined with back propagation neural network, the interlock and residual bottom thickness etc. are optimized through ANN^24^. The SPR molding process can easily lead to various forming defects due to differences in rivet quality, sheet quality differences and equipment stability. Due to the relatively small defects, especially the cracks in the buttons, it is difficult to find by artificial vision, and these defective joints often become the starting point of failure. This is one of the reasons why this paper proposes a new quality monitoring method to solve this limitation. The appearance quality of the joint is mainly detected by means of spot checks, relying on the judgment of engineers. The formed joints with defects are often only a minority, and the sampling method can easily lead to the omission of abnormal joints, leaving a safety hazard, and if the defect is comprehensively checked, it is extremely labor-intensive. In addition, there are no reports in the literature on how to monitor for SPR external defects.

In order to solve the safety mentioned above hazards of SPR defects and further improve the SPR forming quality monitoring system, we propose using visual recognition technology to monitor the external defects of SPR effectively. This paper proposes a non-contact and long-distance non-destructive detection method for SPR defects based on computer vision. Aiming at the actual engineering application scenarios of SPR and the problems of large numbers of deep learning parameters, large amounts of computation, and obstruction of engineering applications. A convolutional neural network algorithm with greater accuracy and less computation is proposed, which accomplishes the optimization of the algorithm’s light weight and accuracy when combined with inverted residual with linear bottleneck, depthwise separable convolution, and adaptive weighting approach.

## Appearance quality monitoring methods

### SPR forming defects and causes of formation

**Figure 3:**
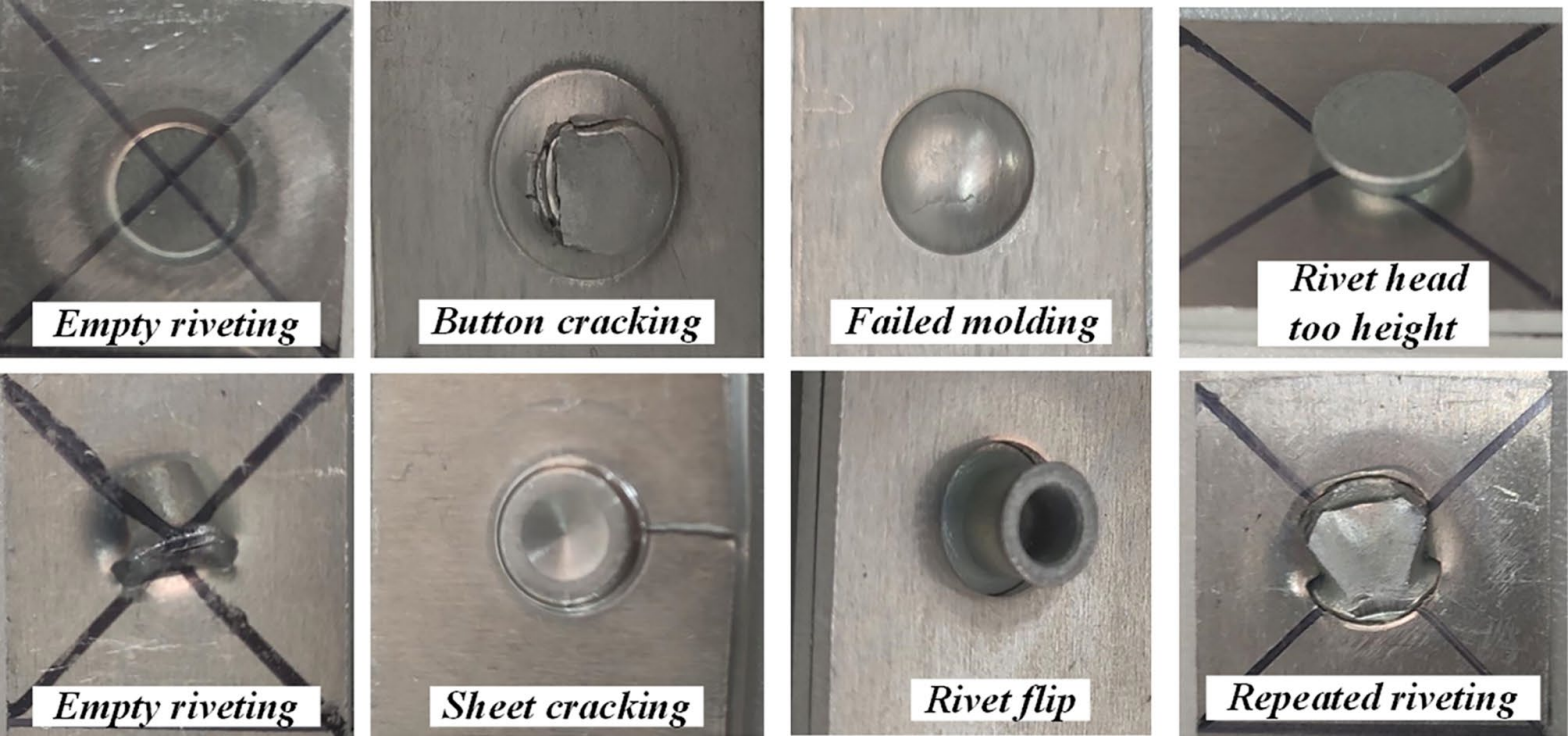
Types of SPR defects.

This paper summarizes the common types of defects in the self-pierce riveting forming process from a large number of formed joints, as shown in Fig. 3. The empty riveting was mainly due to the stuttering of the conveying rivet mechanism, and the rivet cannot be got out normally. As a result, no rivets appeared during the riveting process, and the punch was squeezed directly against the upper sheet to form a rivet-free joint. Repeated riveting is often due to a failure of the rivet feeding mechanism to send out two rivets at the same time, resulting in two rivets stacked on the upper sheet. Sheet cracking is due to individual sheet quality problems or some difference in the local mechanical properties of the sheet caused by cracking in the forming process. Molding failures may be due to fluctuations in the performance of the SPR machine, or failure of the riveting limit that causes the punch to form differently from the preset, thereby affecting the joint forming. Rivet flip due to the reverse of rivet when it is loaded into the feeding mechanism. According to the quality evaluation standards of SPR, the appearance of SPR molding joints is required to have no cracks, full buttons, and the rivet head height and sheet are flush.

### Deep learning algorithms

Traditional image processing algorithms rely on target color and texture features to obtain image feature information, and face a series of challenges such as complex background, defect diversity, brightness, contrast, blur, and resolution variation. Deep learning is not limited to pixel values, and achieves ‘understanding’ of image information in a training manner. Deep learning algorithms are suitable for more complex scenarios, and have stronger generalization capabilities in the face of the changes of background, brightness, target scale, etc.

Deep learning algorithms are mainly for defect processing, mainly object detection, image segmentation, image classification algorithms, etc. The processing process of the image segmentation algorithm needs to traverse all the pixels in the image, and the calculation cost of the algorithm is large and is slow, and it is not suitable for the application of fast riveting scenes. For the image classification algorithm, if two riveting defects occur in the input image at the same time, it is prone to incorrect classification, the field of view is limited, and the defect cannot be located. Compared with the above two class of algorithms, the object detection algorithm is more lightweight and flexible, and can realize the positioning, classification, and counting of the target at the same time, and can realize the detection of multiple targets at the same time. Due to the complexity of the SPR scene, the joint has a high degree of similarity with the background, the riveting range is large and the field of view is large, and the metal surface is susceptible to the influence of light, which brings certain noise to the image. Therefore, the object detection algorithm is more suitable and efficient in the SPR scenario.

### Overview of appearance inspection method

**Figure 4:**
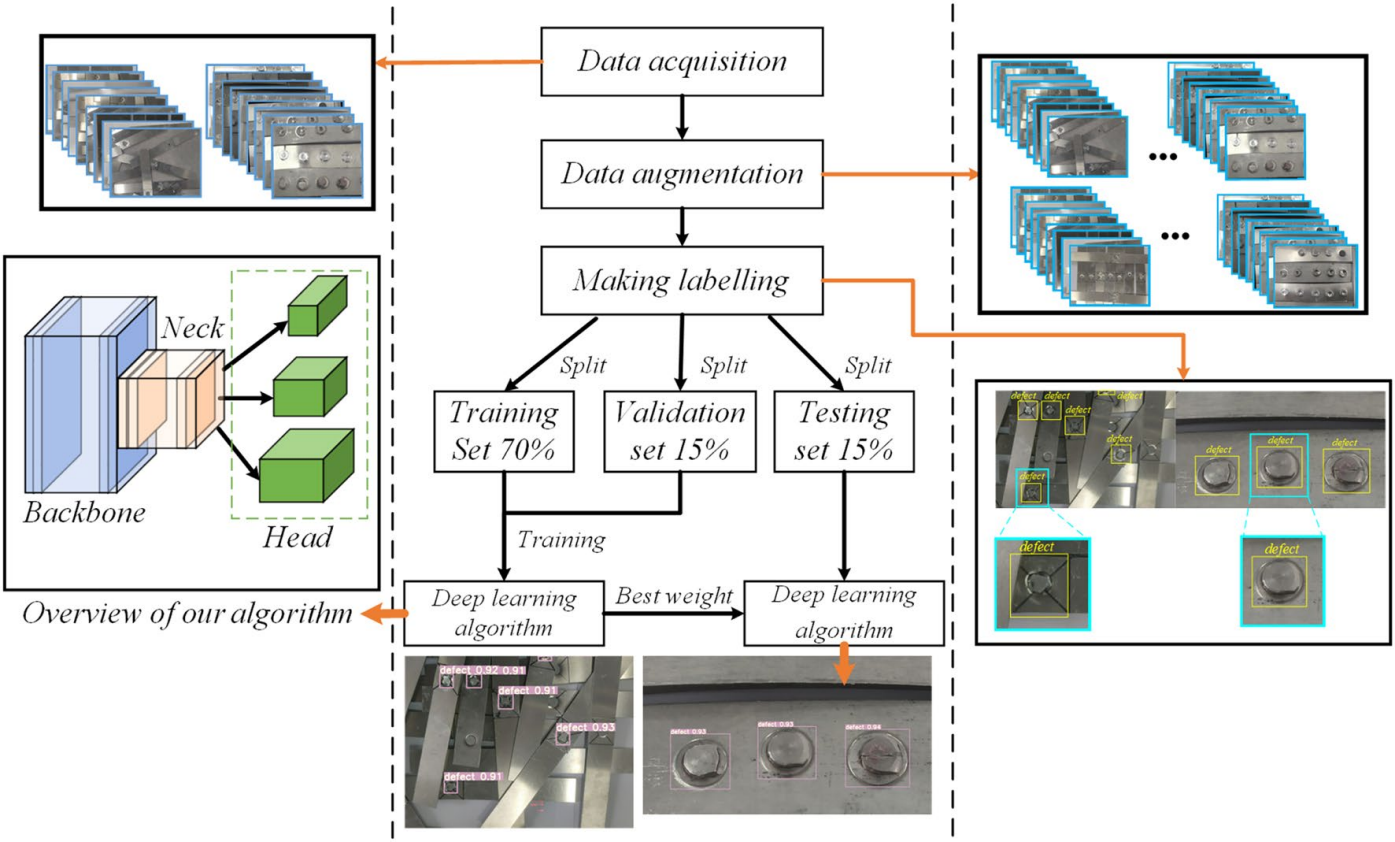
Convolutional neural network realizes SPR quality detection.

In the process of SPR, a variety of appearance quality failures are prone to occur. For this reason, this paper proposes a new non-contact long-distance vision inspection method for the SPR appearance forming quality problem, and the overall implementation process is shown in Fig. 4. First, the image of the molded joint is obtained through a vision sensor. Acquire defective connector images at different backgrounds, resolutions, brightness, and viewing angles. Types of defects include sheet cracking, button cracking, button shedding, repeated riveting, empty riveting, etc. The data enhancement of the acquired images can effectively expand the amount of data while improving the robustness and generalization ability of the algorithm, and ensuring the reliability of the algorithm in different scenarios. The dataset is then randomly divided into training sets, validation sets, and testing sets, and corresponding annotation files are made. Finally, the algorithm is trained, verified and tested, and the performance of the algorithm is measured by evaluation indicators.

## Methodology

**Figure 5:**
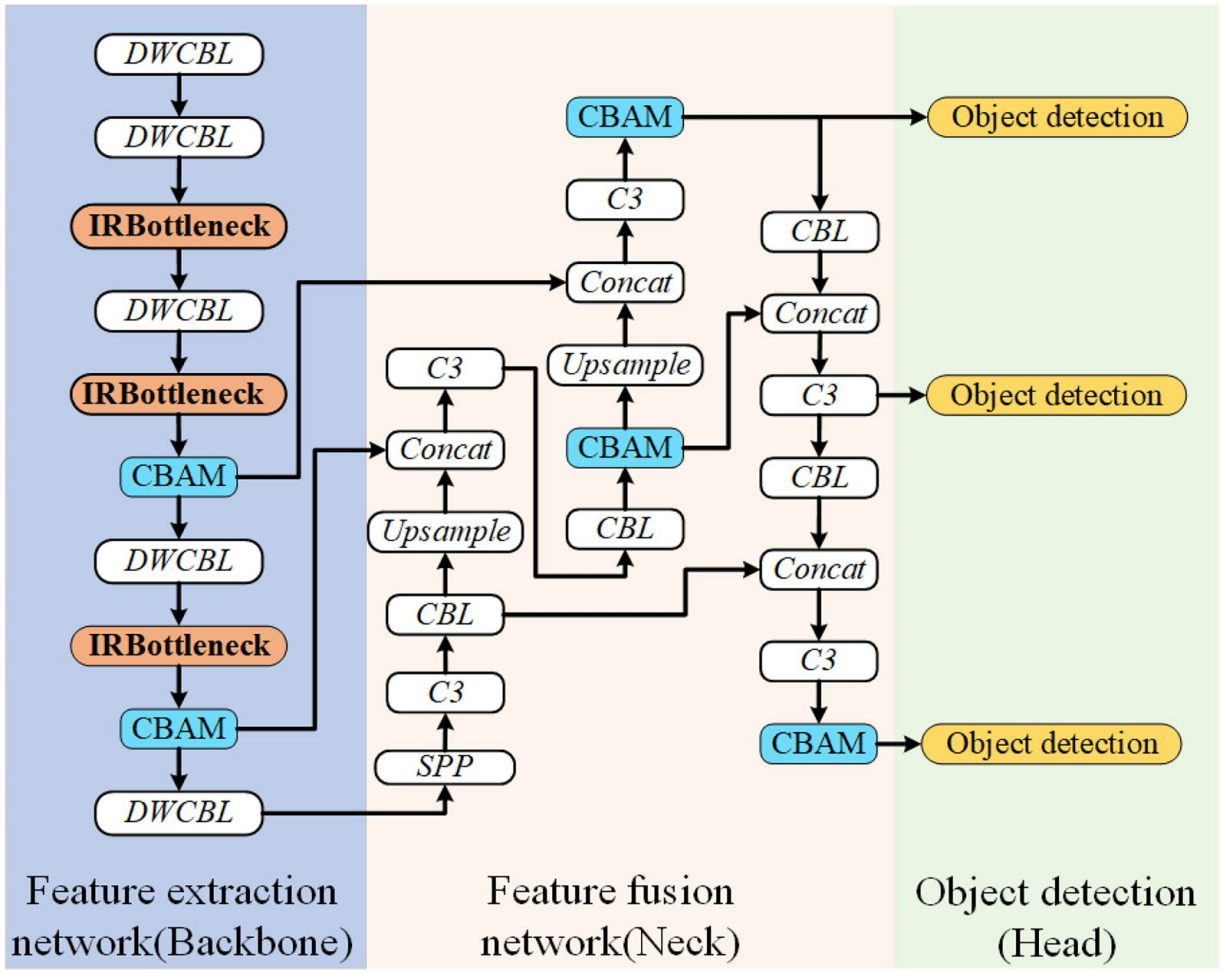
The overall architecture of the proposed algorithm.

In this paper, we combine deep learning algorithms to monitor the quality of SPR, and optimize the feature extraction network and feature fusion network based on the Yolov5^25^ algorithm for SPR application scenarios to ensure high detection accuracy and low computational overhead. The framework of the proposed algorithm consists of three structures: feature extraction network (Backbone), feature fusion network (Neck) and object detection (Head). Among them, the Backbone is composed of the inverted residual with linear bottleneck (IRBottleneck), depthwise separable convolution (DWCBL), convolutional block attention mechanism (CBAM) and spatial pyramid pooling (SPP), while the Neck is composed of traditional convolutional blocks, feature multiplexing modules (C3) and CBAM, and object detection follows the mesh positioning method of Yolov5 algorithm. The overall architecture of the algorithm in this paper is shown in Fig. 5.

### Depthwise separable convolution

**Figure 6:**
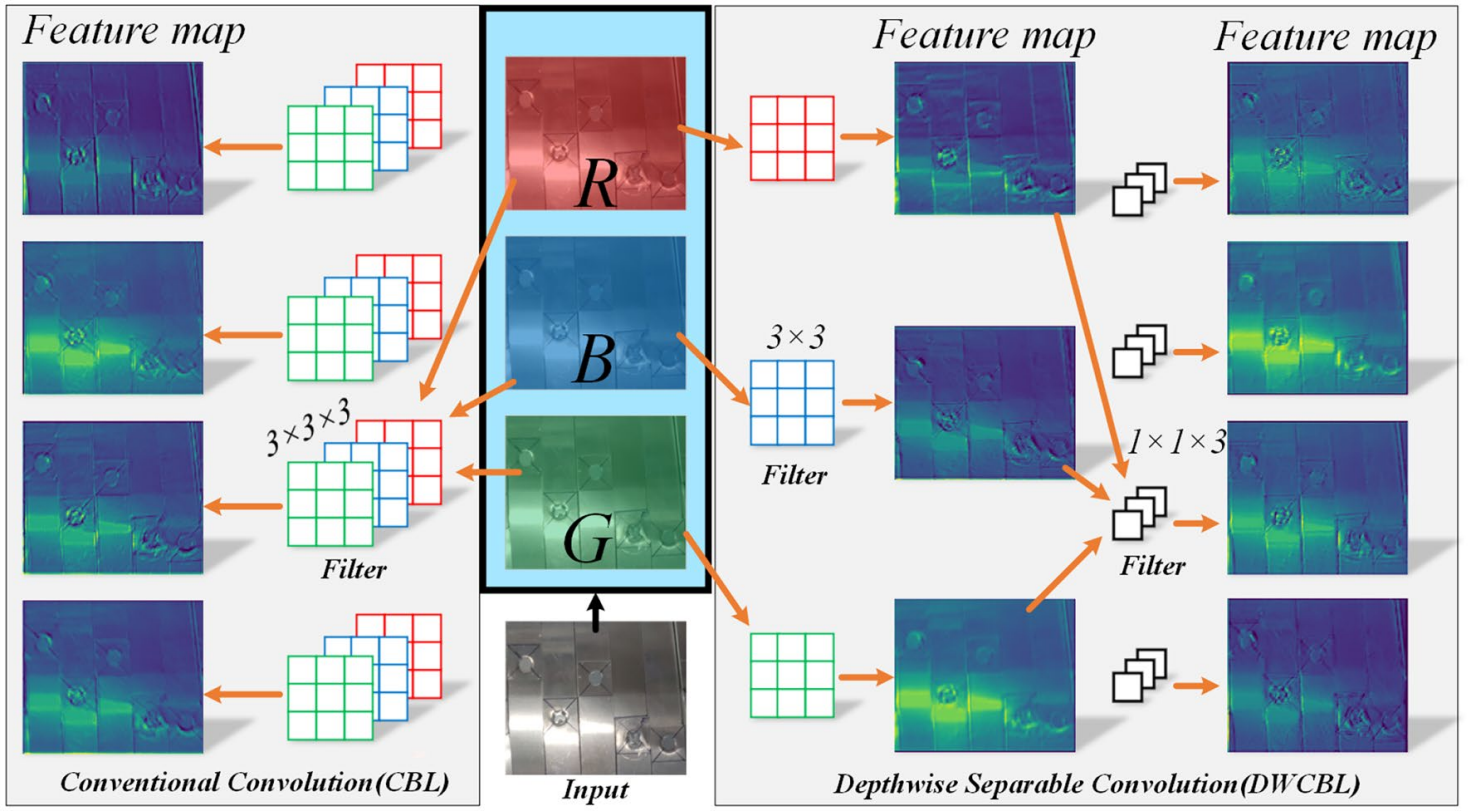
Depth separable convolution differs from conventional convolution.

In order to efficiently extract the underlying feature information and reduce the number of redundant parameters and computation, Backbone is combined with depthwise separable convolution. Depthwise separable convolution can effectively achieve on convolutional layer acceleration, Xception^26^, MobileNetV1^27^ and other algorithms have achieved algorithm lightweighting by combining depthwise separable convolution. Using this new convolution operation, replacing the traditional convolutional structure can effectively reduce the amount of computation and parameter. The underlying features are extracted primarily by the Backbone. The GhostNet experimental findings reveal that the shallow feature layers have relatively comparable characteristics, and that the shallow features have a linear relationship^28^. Combined with depthwise separable convolution can simultaneously achieve the extraction of underlying features and the depth of the extended network, and at the same time, can ensure a certain linear relationship between shallow features. The difference between depthwise separable convolution and conventional convolution is shown in Fig. 6. When the dimensions of the input and output are the same, the depthwise separable convolution is equivalent to splitting the conventional convolution into two steps. First by depthwise convolution and then by pointwise convolution to adjust the number of channels. Taking Fig. 6 as an example, the parameters of conventional convolution are $4 \times 3 \times 3 \times 3=108$, while the total parameter amount of depthwise separable convolution using depth is 39, of which the parameter amount of depthwise convolution is $3 \times 3 \times 3$, and the parameter of pointwise convolution is $1 \times 1 \times 3 \times 4=12$. The parameter amount of depthwise separable convolution is 1/3 of that of conventional convolution. The reduction of the amount of parameters can effectively reduce the display pressure, calculation pressure and communication pressure of ordinary equipment.

### A lightweight strategy for IRBottleneck

AlexNet^29^ has aroused a wave of application of convolutional neural networks in various fields due to its amazing performance on the ImageNet dataset. Various algorithms with higher accuracy have appeared one after another, such as Single shot multibox detector(SSD)^30^, CenterNet^31^, etc. However, the performance improvement obtained by building more complex, deeper, and wider convolutional layers requires higher computing resources. These algorithms often exceed the computing power of most mobile and embedded devices, and devices with high computing power become the threshold for algorithm application.

**Figure 7:**
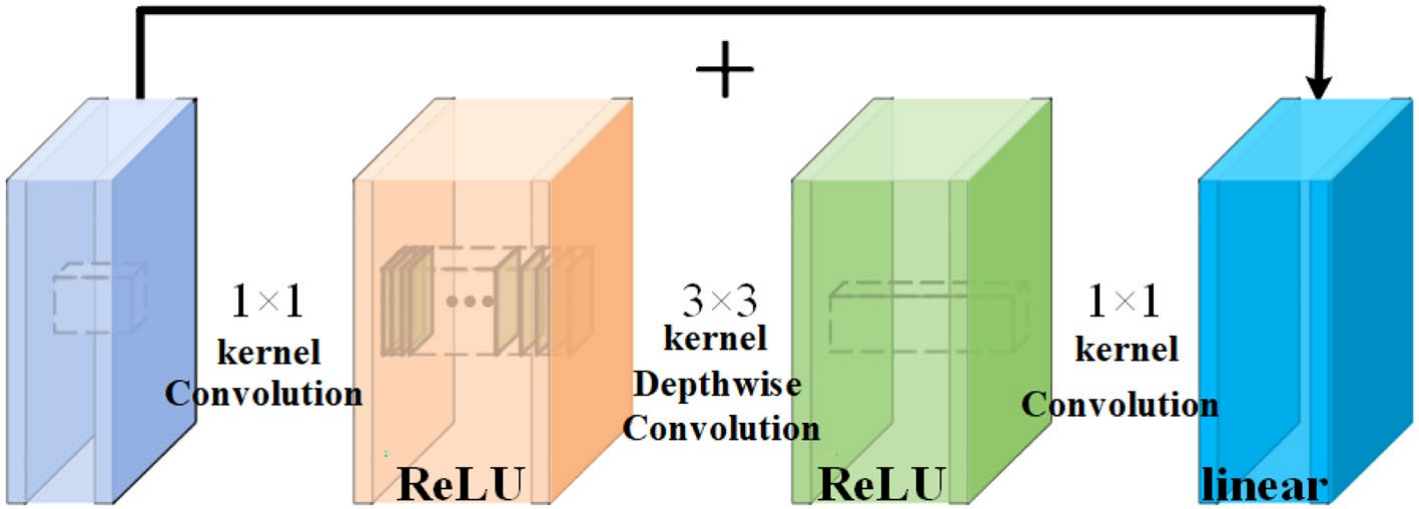
IRBottleneck.

In order to achieve the balance between algorithm precision and computational consumption, a new mobile framework in the field of computational vision, namely IRBottleneck, is used to construct Backbone, which can be used on mobile devices and various tasks^32^. The IRBottleneck structure is shown in Fig. 7. First, the input low-dimensional features are expanded to high-dimensional space through $1 \times 1$ convolution, and deepening the structure of the intermediate layer can minimize the loss of information during the transfer process. The higher the output dimension of the transfer layer, the less information is lost. The features are further extracted using depthwise convolutional filters, and finally the features are mapped back to the low-dimensional space again through $1 \times 1$ convolution and linear activation function. The information flow of the region of interest (ROI) is stored in some channels in the layer structure. By reducing the dimension of the operation space, the information of these ROI can be effectively captured and utilized. And a linear activation function is used in the final output of the module, which effectively avoids the collapse of feature information and leads to information loss. This structure is suitable for mobile devices, reduces the calculation amount of the algorithm inference process, and significantly reduces the memory occupation of the device.

### Adaptive weighting strategy

Attention is a simple and efficient feature-adaptive reinforcement mechanism, and the attention mechanism is widely used in natural language processing^33^, speech recognition^34^, object detection and classification^35^, etc. To understand attention from a more essential perspective, the attention mechanism is to assign higher weights to more important target information, and to assign less weight to unimportant information, so as to allocate computing resources reasonably. It can be seen from the experimental results of many algorithms on public dataset^36,37^ that combining the attention mechanism can better handle complex image information and improve the algorithm’s understanding of the ROI.

**Figure 8:**
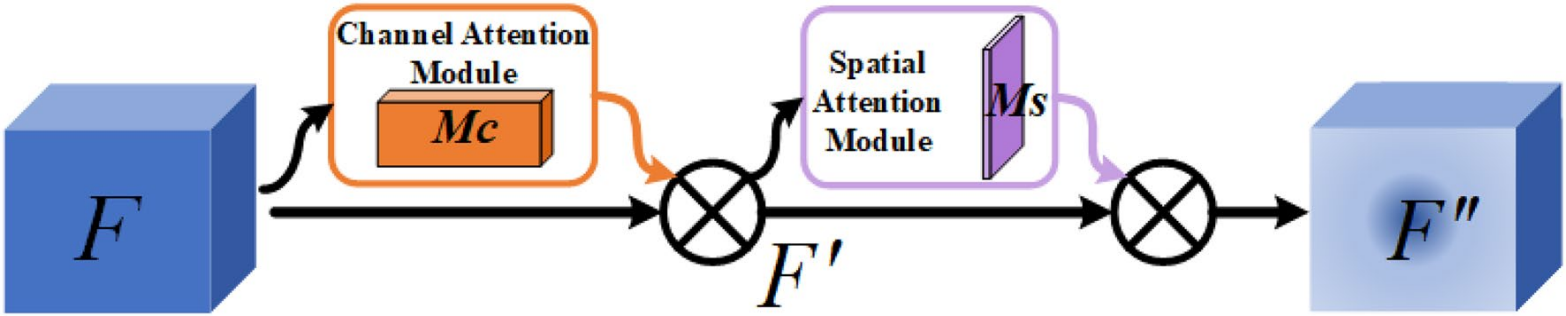
Convolutional block attention mechanism.

The lightweight process of the algorithm often leads to a decrease in performance. At the same time, with the deepening of the convolution layer and the use of a large number of downsampling, the target information will be lost to a certain extent, especially for small targets or targets similar to the background impact is more serious. The algorithm in this paper utilizes the CBAM in the Backbone and Neck to enhance the feature information from the channel and space as a whole. Using CBAM can effectively strengthen target information, weaken unnecessary background information, and effectively improve the representation of ROI. It can effectively improve the performance of the algorithm with very little parameter amount and computational overhead. The overall architecture of CBAM is shown in Fig. 8.

CBAM mainly contains two structures, namely channel attention and spatial attention. Each channel of channel attention is equivalent to a filter, which can strengthen the high-level semantic information in the channel, and the main role of spatial attention is to effectively enhance the contour representation of the ROI. The overall implementation mechanism of CBAM is: feature mapping for an input $F\in R_{C\times H\times W}$. The one-dimensional channel attention map $M_{c}\in R_{C\times 1\times 1}$ is obtained through the channel attention module, and the $F{}'$ is obtained by multiplying with the input feature, and the two-dimensional spatial attention map ($M_{s}\in R_{1\times H\times W}$) is obtained by using the spatial attention module, and finally $F{}'$ is multiplied with Ms to obtain $F{}''$, the above calculation formula is as follows: Eqs. (1) and (2).

1 $$\begin{aligned} F{}'= & {} M_{c}\left( F \right) \otimes F \end{aligned}$$

2 $$\begin{aligned} F{}''= & {} M_{s}\left( F{}' \right) \otimes F {}' \end{aligned}$$

**Figure 9:**
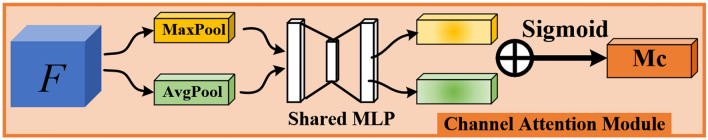
Channel attention module.

The way to make a channel attention map using input features is shown in Fig. 9. First, average pooling and max pooling are performed along the spatial direction to aggregate the channel information of each feature. Then add them through the shared multi-layer perceptron(MLP) network respectively, and finally obtain the channel attention map through the sigmoid function. The acquisition principle of the channel attention map is as shown in formula (3).

3 $$\begin{aligned} M_{c}\left( F \right) =S\left( MLP\left( AvgPool\left( F \right) \right) +MLP\left( MaxPool\left( F \right) \right) \right) \end{aligned}$$

**Figure 10:**
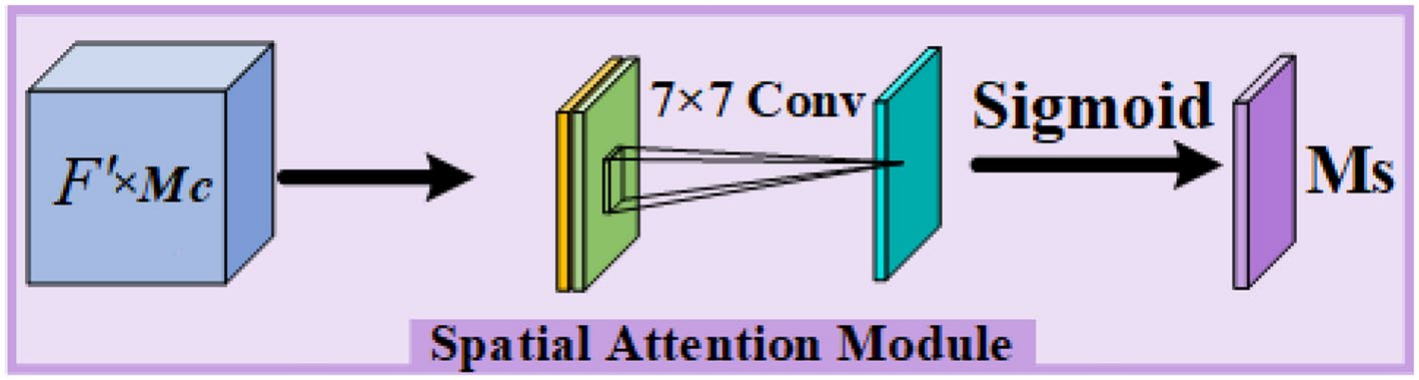
Spatial attention module.

Spatial information mainly includes target position and contour information, combined with the spatial attention map, it can effectively strengthen the expression of the area of interest. The acquisition method of the two-dimensional spatial attention map is shown in Fig. 10. The maximum pooling and average pooling are performed along the channel direction respectively, and the results of the two pooling are spliced and compressed and fused by $7 \times 7$ convolution, and finally converted into weight values by the sigmoid function.

## Experimental verification

### Dataset and experimental environment

**Figure 11:**
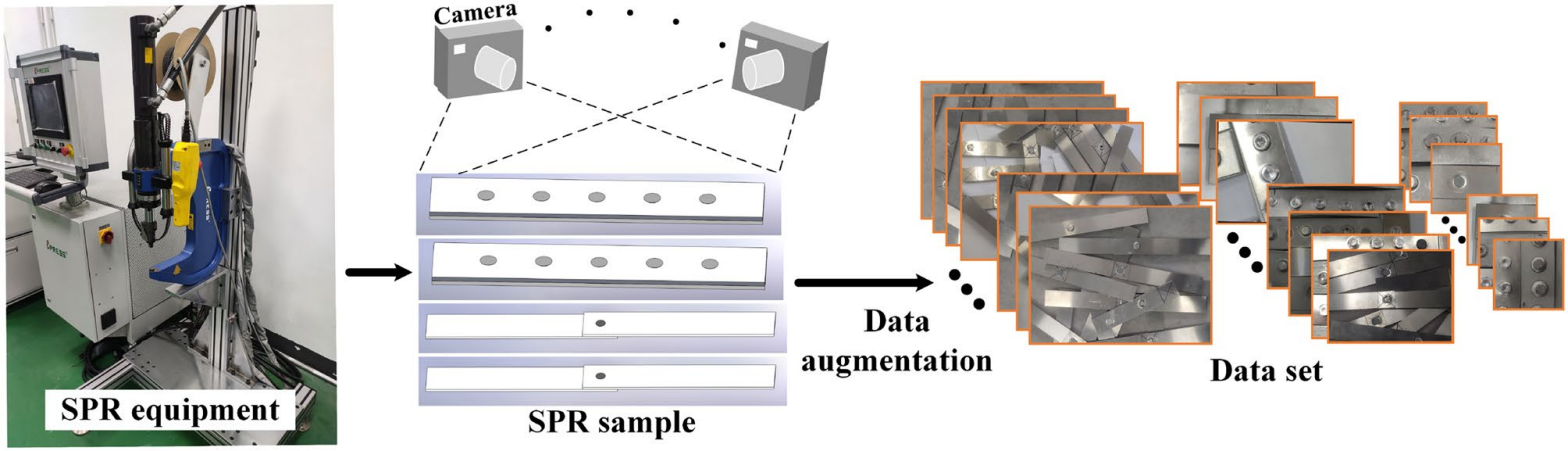
Experimental equipment, specimen types, and dataset construction.

Obtaining a large number of defective riveted joints directly on the automobile body will consume a lot of manpower and material resources, and is too expensive, which is impractical in terms of time and cost. For this reason, standard riveted joints are made by referring to the sampling method of GB 2649-89 welded joint mechanical performance test, and the riveting situation with a large interval and a large range is simulated. At the same time, select a part of the sheet for riveting in a denser way to simulate the scene of dense riveting. The SPR experimental platform and visualization part of the dataset are shown in Fig. 11. While increasing the amount of data, it also tests the actual performance of the algorithm.

Due to the characteristics of the SPR forming process, the differences in the shape of different joints after SPR forming are relatively small. Similarly, the feature differences of the same type of defects are relatively small. Therefore, in the process of constructing the dataset, under the premise of having an adequate amount of data, it is necessary to ensure the completeness of the defect types and the diversity of the data to fit the network model well. Therefore, images of all possible defect types that may appear in the SPR process were collected under different scenes, including backgrounds, brightness, resolution, and stacking of riveting parts. The defect types include cracks in thin plates, button cracks, forming failures, rivet flipping, empty rivets, double rivets, and high rivet heads. A total of 242 original images were collected. Then, data augmentation methods such as rotation, brightness variation, and color transformation were used to expand the data and increase the diversity of the dataset, resulting in 434 images in total. Data augmentation, as a cheap way to expand data, can effectively avoid overfitting, reduce sensitivity to data, and improve the robustness and generalization ability of the algorithm^38^.

The 434 images obtained were used to label the data labeling software Labelimg to make training set, validation set, and testing set, with 70$\%$, 13$\%$, and 17$\%$ respectively. Assign more and harder to detect data to the testing set than to the validation set. To ensure fair comparisons between algorithms, all algorithmic environments were configured on a desktop (Intel(R) Core (TM) i7-10700k CPU @3.80GHz x16 and GeForce RTX 2080 SUPER GPU) with a training epoch of 500, a batch-size of 6, and the rest of the parameters were kept default.

### Ablation studies overview

This paper will systematically explore the influence of IRBottleneck, depthwise separable convolution and attention mechanism on the performance of the algorithm through ablation experiments, so as to design a more efficient algorithm. Ablation experiments will be conducted from the following perspectives, respectively, by validating MoblileNetV2 as the Backbone of the Yolov5 algorithm (+MoblieNet); IRBottleneck combined with conventional convolution as the Backbone (+IRB+C); IRBottleneck combined with depthwise separable convolution as the Backbone (+IRB+D); the IRBottleneck combined with depthwise separable convolution is used to construct the Backbone, and then the attention mechanism is introduced on the Backbone and Neck as proposed in this paper (+IRB+D+A(Our)). The specific ablation experiments are shown in Table 1. At the same time, it is compared with algorithms such as SSD, CenterNet, and Yolov5.

**Table 1:** Ablation studies.

Algorithm	Backbone					Neck
	MoblieNetV2	IRBottleneck	CBL	DCBL	CBAM	CBAM
+MoblieNet	$\checkmark$	$\times$	$\times$	$\times$	$\times$	$\times$
+IRB+C	$\times$	$\checkmark$	$\checkmark$	$\times$	$\times$	$\times$
+IRB+D	$\times$	$\checkmark$	$\times$	$\checkmark$	$\times$	$\times$
+IRB+D+A(Our)	$\times$	$\checkmark$	$\times$	$\checkmark$	$\checkmark$	$\checkmark$

All algorithms are experimented on the same dataset, and the loss function allows to analyze the error between the predicted and ground truth of the algorithm training process. The classification loss function for training uses the Binary Cross Entropy loss function, which is used to measure the accuracy of sample classification, and its expression is shown in Eq. (4), and the bounding box regression uses the $L_{CIoU}$ function^39^, whose expressions are shown in Eqs. (5)–(7).

4 $$\begin{aligned} L=-\sum _{i=1}^{N}y^{\left( i \right) }log {\hat{y}}^{\left( i \right) }+\left( 1-y^{\left( i \right) } \right) log\left( 1-{\hat{y}}^{\left( i \right) } \right) \end{aligned}$$

${\hat{y}}$ represents the probability that the algorithm predicts that the i-th sample is a certain class, $y^{\left( i \right) }$ represents the label, and N represents the total number of samples.

5 $$\begin{aligned} \Re _{CIoU}= & {} \frac{\rho \left( b,b^{gt} \right) }{c^{2}}+\alpha V \end{aligned}$$

6 $$\begin{aligned} V= & {} \frac{4}{\pi ^{2}} \left( arctan\frac{w^{gt}}{h^{gt}}-arctan\frac{w}{h} \right) ^{2} \end{aligned}$$

7 $$\begin{aligned} L_{CIoU}= & {} 1-IoU+\Re _{CIoU} \end{aligned}$$

$\rho$ denotes the distance between the two points of the solution, *b* and $b^{gt}$ denote the centroids of the prediction bounding box and the ground truth box, respectively. *c* denotes the minimum diagonal length of the two bounding boxes, $\alpha$ is the equilibrium parameter. $\alpha$ is 0 when intersection over union (IoU) is less than 0.5, and $\alpha$ is $V/(1-IoU)+V$ when IoU is higher than 0.5. The similarity of the aspect ratio is measured by *V*, and the width-height information is considered in the regression process of the bounding boxes. $w^{gt}$, $h^{gt}$ and *w*, *h* denote the width and height of the ground truth box and the prediction bounding box, respectively.

**Figure 12:**
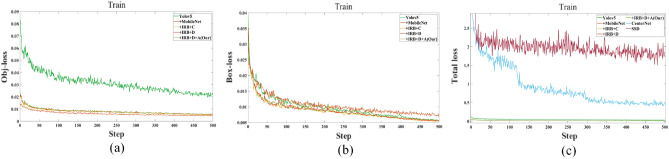
Results of training.

The training results are shown in Fig. 12. In addition, the impact of the optimization module on the classification loss and bounding box loss will be analyzed through the loss value. Fig. 12a is the object classification loss, Fig. 12b is the bounding box regression loss, and Fig. 12c is the total loss. After the network training, it can be seen from the analysis of the classification loss, bounding box loss and total loss of training that the classification loss value of all algorithms has reached a small value, and it can be seen from Fig. 12a that a IRBottleneck is added to the algorithm, which can make the algorithm have better convergence and quickly reach a smaller loss value. After algorithm training, all algorithms are tested.

### Algorithm performance evaluation metrics

**Figure 13:**
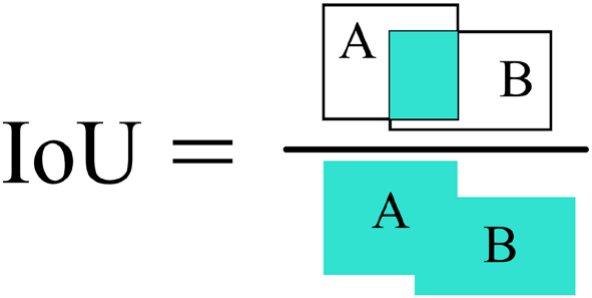
The calculation method of the IoU of the two bounding boxes.

This paper will evaluate the algorithm performance, space complexity, and time complexity through multiple dimensions such as precision, recall, mean average precision (mAP), Giga floating point operations per second (GFLOPs), and the number of model parameters, where precision is used to measure the detection accuracy of the algorithm. The algorithm’s capacity to locate all positive classifications is measured by recall, which is used to assess the algorithm’s degree of missed detection. The greater the recall, the less likely it is to be missed. GFLOPs is a general indicator used to measure model complexity, and it is also commonly used to measure the speed of neural network models. The larger the GFLOPs, the more complex the model, the more computing resources the model occupies, and the higher the device performance requirements. The formula for precision and recall are shown in Eqs. (8) and (9). The formula for mAP is shown in Eqs. (10) and (11). Because there is only one category in this paper, average precision (AP) is equal to mAP. The mAP is different under different IoU thresholds. The IoU is calculated as shown in Fig. 13.

8 $$\begin{aligned} Precision= & {} \frac{TP}{TP+FP} \end{aligned}$$

9 $$\begin{aligned} Recall= & {} \frac{TP}{TP+FN} \end{aligned}$$

10 $$\begin{aligned} AP= & {} \int _{0}^{1}P\left( R \right) d\left( R \right) \end{aligned}$$

11 $$\begin{aligned} mAP= & {} \frac{1}{N}\sum _{N}^{1}\int _{0}^{1}P\left( R \right) d\left( R \right) \end{aligned}$$

Positive samples are correctly classified as true positive, denoted as *TP*, and positive samples are incorrectly classified as false negative, denoted as *FN*. Negative samples are correctly classified as true negative, denoted as *TN*, and negative examples are incorrectly classified as false positive, denoted as *FP*. *P* means precision, *R* means recall, and *N* means the number of categories.

### Quantitative analysis of algorithm performance

The algorithms mentioned above and comparative algorithms for ablation experiments are tested to analyze the performance of different algorithms comprehensively. Table 2 shows the quantitative comparison results of different algorithms. Precision, recall, $mAP_{0.5}$, parameters, and GFLOPs are selected to analyze the algorithm’s overall performance. The verification performance reflects the learning ability of the algorithm to the data to a certain extent, and the test performance shows the application effect of the algorithm, reflecting the strength of the general ability of the algorithm.

**Table 2:** Qualitative comparison of algorithms.

Algorithm	Experiment	$Map_{0.5}$	Testing		Parameters/k	GFLOPs
			Precision	Recall		
Yolov5	1	96.7	84.7	74.5	46631	114.1
+MoblieNet	2	92.7	90.5	66.8	5959	41.5
+IRB+C	3	96.8	86.7	75.3	7770	72.1
+IRB+D	4	97.0	88.5	70.5	6225	60.0
+IRB+D+A(Our)	5	97.1	89.2	75.9	6274	60.1
SSD	6	89.7	76.3	55.0	23750	342.75
CenterNet	7	40.1	52.6	63.8	14210	51.01

It can be seen from Table 2 that the network constructed by integrating the MoblieNet architecture (Experiment 2) can effectively reduce the number of parameters, which is 87$\%$ lower than the original Yolov5 algorithm, and the computational cost is reduced by 63.6$\%$. Moreover, the precision has been greatly improved. However, the overall performance of +MobileNet has dropped significantly. Its $mAP_{0.5}$ dropped by 4$\%$, respectively, and the recall dropped by 7.7$\%$. The direct combination of the MoblieNet achieves a significant reduction in the number of parameters and computation, thus achieving the maximum weight reduction of the algorithm. However, the significant reduction in the number of parameters of the feature extraction network and the too small channel of the feature extraction network make the memory capacity of the feature extraction network drop rapidly, which may easily lead to a significant drop in the overall performance.

The analysis results of experiments 3 and 4 show that using the linear inverted residual module and standard convolution or depthwise separable convolution to build a new backbone can effectively reduce the number of algorithm parameters and computational overhead at the same time, and the performance of the algorithm in verification both are very close to the Yolov5 algorithm. This strategy can effectively improve the recall and $mAP_{0.5}$, and the performance improvement is more apparent when combined with traditional convolution, but traditional convolution also introduces more parameters and calculations.

Experiment 5 is combined with an adaptive weighting strategy based on experiment 4. From the results of experiment 5, it can be seen that combining CBAM can effectively improve the recall and precision of the test. At the same time, the number of parameters introduced and the amount of computation can be negligible. Fusion CBAM can achieve the simultaneous improvement of improving each performance index while ensuring the lightweight of the algorithm. The analysis of the experimental results of SSD and CenterNet (Experiments 6 and 7) shows that the precision, recall, and $mAP_{0.5}$ of both are relatively low. SSD and CenterNet perform poorly in scenarios where the target is occluded, the background is highly similar to the target itself, and the target scale is variable. In addition, compared with the algorithm in this paper, the number of parameters and computation of both are significant.

The algorithm in this paper achieves both accuracy improvement and algorithm lightweight. Compared with Yolov5, the precision is increased by 4.5$\%$, the recall rate is increased by 1.4$\%$, and $mAP_{0.5}$ is increased by 0.4$\%$. In addition, the redundant parameters are reduced by 86.5$\%$, and the calculation amount is reduced by 47.33$\%$. Compared with experiments 3–4, the increased parameters and computational overhead are negligible.

### Qualitative comparison

**Figure 14:**
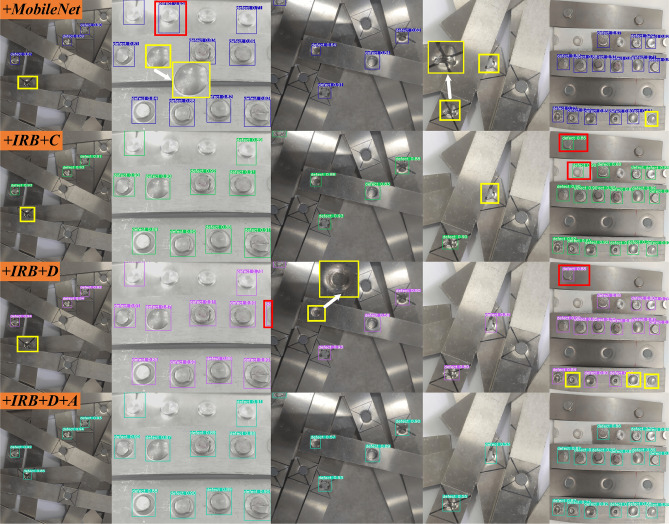
Qualitative comparison results of different improved methods, each row represents the detection result of one algorithm. Missed and incorrectly detected targets are marked with yellow and red rectangles, respectively.

Table 2 systematically analyzes the performance of all algorithms, that is, the comprehensive performance of the algorithm. This chapter will further analyze the actual detection effect of the algorithm through a qualitative comparison algorithm. The detection effect of the algorithm related to the ablation experiment is shown in Fig. 14. Each row represents the detection results of each algorithm, with missed detections and false detections marked with yellow and red rectangles, respectively. The overall analysis shows that although all the comparison algorithms can detect riveting defects, they have a certain degree of missed or false detection. Because the target resolution is large or the target and the background are similar, +MobileNet is prone to missed detection. +IRB+C and +IRB+D have more false detections and a small number of missed detections, to a certain extent, because of the reduction in the number of model parameters. From the detection results of +IRB+D+A(Our), it can be found that adaptive weighting can reduce false detection and missed detection, which is consistent with the quantitative analysis results.

**Figure 15:**
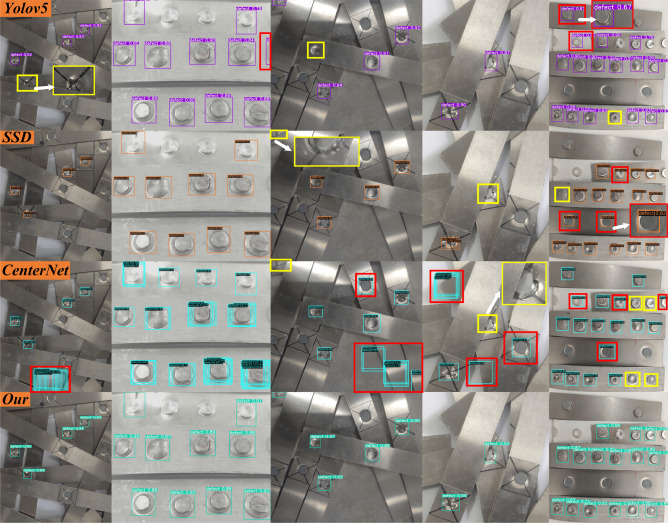
Qualitative comparison of different algorithms. Each row represents the detection results of one algorithm, from top to bottom, Yolov5, SSD, CenterNet, and Our. Missed and incorrectly detected objects are marked with yellow and red rectangular boxes, respectively.

A qualitative comparison of the different algorithms is shown in Fig. 15. CenterNet has the most missed detections and errors among all the comparison algorithms, and there is a problem with multiple detection boxes for one target. SSD is easy to miss detection of occluded targets, and when the shape of the target is similar to the background, SSD is prone to misidentification. When the target is blurry or highly similar to the background, the Yolov5 algorithm will cause misrecognition. The method in this paper can effectively reduce the number of model parameters and calculations, maximize the detection accuracy, and reduce the missed detection rate and false detection. From the qualitative comparison results, it can be concluded that the method in this paper not only has high detection precision, but also can achieve better robust detection in scenes such as occlusion, scale change, blur, and brightness change.

## Conclusion

In this paper, an end-to-end deep learning algorithm is proposed to supervise the appearance quality of SPR, and the Backbone and Neck are optimized for the application scenarios of SPR technology. By combining the IRBottleneck and the depthwise separable convolution, the algorithm is lightweight, and the completeness of the target information in the feature extraction process is guaranteed while reducing the amount of parameters and computation. The adaptive weighting strategy is used to realize the adaptive weighting of the key feature information in the information flow, which effectively strengthens the characterization of the ROI. Ablation experiments verify the accuracy and rationality of the proposed algorithm. The experimental results show that the algorithm’s detection precision, recall , and $mAP_{0.5}$ are improved by 4.5$\%$, 1.4$\%$, and 0.4$\%$, respectively, and the calculation amount is reduced by 47.33$\%$. The algorithm in this paper improves the detection accuracy while effectively reducing the missed detection rate, false detection, memory usage, and computational complexity.

The main contributions of this paper can be summarized as follows: (1) Change the traditional manual visual method, and propose a method based on computer vision to realize intelligent monitoring of external defects of SPR, which can effectively monitor SPR defects such as double riveting, sheet cracking, empty riveting, and button micro-cracks. (2) The inverted residual with linear bottleneck is proposed to realize the optimization of the algorithm, which significantly reduces the amount of calculation and parameters, making the algorithm more conducive to deployment and application in engineering. (3) An adaptive weighted optimization method is proposed, which can effectively improve the precision and recall of the algorithm under the condition of negligible parameters and computational overhead.

SPR involves numerous process parameters, including pre-tightening force, puncture force, holding force, stroke, and die. In most cases, there are multiple sets of optional parameters available for connecting thin sheet materials, but only one set of parameters will produce the least amount of defects. Algorithms can be used to detect and tally the types and quantities of defects under different process parameters, thereby achieving optimization of the process parameters. Furthermore, our approach can be used in conjunction with the curve tolerance band method to construct a more efficient SPR defect detection system, providing a new method for improving the SPR supervision system.

## Data Availability

The datasets generated during and/or analysed during the current study are not publicly available due to ongoing development of the algorithms but are available from the corresponding author on reasonable request.
